# Diabetes and Neuroaxonal Damage in Parkinson's Disease

**DOI:** 10.1002/mds.29067

**Published:** 2022-07-20

**Authors:** Nirosen Vijiaratnam, Michael Lawton, Raquel Real, Amanda J. Heslegrave, Tong Guo, Dilan Athauda, Sonia Gandhi, Christine Girges, Yoav Ben‐Shlomo, Henrik Zetterberg, Donald G. Grosset, Huw R. Morris, Thomas Foltynie, PRoBaND Clinical Consortium

**Affiliations:** ^1^ Department of Clinical and Movement Neurosciences UCL Queen Square Institute of Neurology London United Kingdom; ^2^ School of Social and Community Medicine University of Bristol Bristol United Kingdom; ^3^ Department of Social Medicine University of Bristol Bristol United Kingdom; ^4^ Aligning Science Across Parkinson's (ASAP) Collaborative Research Network Chevy Chase Maryland USA; ^5^ Dementia Research Institute University College London London United Kingdom; ^6^ Department of Neurodegenerative Disease UCL Institute of Neurology, Queen, Square London United Kingdom; ^7^ Clinical Neurochemistry Laboratory Sahlgrenska University Hospital Mölndal Sweden; ^8^ Department of Psychiatry and Neurochemistry Institute of Neuroscience and Physiology, The Sahlgrenska Academy at the University of Gothenburg Mölndal Sweden; ^9^ Hong Kong Center, for Neurodegenerative Diseases Hong Kong People's Republic of China; ^10^ Department of Neurology, Southern General Hospital University of Glasgow and Institute of Neurological Sciences Glasgow United Kingdom

We read with interest Uyar and colleagues' recent report on the association between diabetes, nondiabetic elevated glycated hemoglobin levels (HbA1c), and neuroaxonal damage in Parkinson's disease (PD) patients from the MARK‐PD study.^1^ The authors confirmed previously established findings of an inverse association between diabetes and cognitive and motor status. The authors also demonstrated higher serum neurofilament light (NfL) levels (a marker of neuroaxonal damage)^2^ in PD patients with prevalent type 2 diabetes and in PD patients with nondiabetic elevated HbA1c levels. These associations persisted after adjustment for age, body mass index (BMI), and vascular risk factors (prevalent arterial hypertension, hypercholesterolemia, and history of stroke). We recently noted similar motor and cognitive associations in PD patients with diabetes^3^ in the Tracking Parkinson's study, although only a nonsignificant trend toward an association in the overall PD cohort between NfL levels and more severe motor and cognitive status at baseline,^4^ which may reflect the reduced disease duration in the Tracking Parkinson's cohort, compared with the MARK‐PD cohort.

Considering the authors' novel findings of an association between diabetes and neuroaxonal damage, we explored the relationship between serum NfL and diabetes in our previously defined subgroup of the Tracking Parkinson's study.^4^ The analysis was performed using Stata V.17.0 (Stata, RRID:SCR_012763), and differences were compared using Kruskal–Wallis tests for continuous data and χ2 tests for categorical data, whereas the association between NfL and diabetes was further explored using univariate and multivariate (age, BMI, and vascular risk factors) linear regression analysis.

Of the 280 patients studied, 29 suffered from prevalent type 2 diabetes. PD‐DM patients were older (74.1 years ± SD 7.7 vs. 68.1 years ± 8.7, *P* < 0.001), with higher BMIs (31.1 ± SD [standard deviation] 5.7 vs. 27.1 ± SD 4.4, *P* < 0.001), whereas a higher proportion had coexistent vascular risk factors than PD patients without diabetes (*P* = 0.032). Serum NfL levels were higher in PD‐DM patients (39.5 ± SD 18.9 vs. 29.6 ± SD 16.0, *P* < 0.001). Using regression analysis, NfL levels were significantly associated with patients' diabetic status (coefficient: 0.82, 95% CI [confidence interval]: 0.45–1.19, *P* < 0.0001), which persisted (coefficient: 0.52, 95% CI: 0.18–0.86, *P* = 0.003) after adjustment for age, BMI, and vascular risk factors (history of angina, myocardial infarction, stroke, hypertension, and hypercholesterolemia).

Our findings affirm Uyar et al's report of an association between PD‐DM and more severe neuroaxonal damage. Furthermore, the data indicate that the more severe phenotype in PD‐DM noted to date by several studies is likely to be mediated by additional factors other than vascular risk factor burden that tends to coexist in these cases. T2DM and PD share several pathological processes encompassing neuroinflammation, lysosomal dysfunction, mitochondrial dysfunction, and the development of central insulin resistance that leads to neurodegeneration.^5^ This process is in part mediated by hyperglycemia as demonstrated by the MARK‐PD study and its downstream impact on α‐synuclein aggregation.^6^ It is also possible that some of the observed associations are explained by diabetic neuropathy, as other peripheral neuropathies are known to increase blood NfL concentrations.^7^ Disentangling the mechanistic factors that contribute to this more rapidly progressive axonal damage is of critical importance in the development of disease‐modifying therapies for PD.

## Full financial disclosures for the previous 12 months

N.V. has received unconditional educational grants from Ipsen and Biogen; travel grants from Ipsen, AbbVie, and the International Parkinson's Disease and Movement Disorders Society; and speaker's honorarium from AbbVie and Stada and served on advisory boards for AbbVie and Brittania outside of the submitted work.

M.L. has no competing interest.

R.R. has no competing interest.

A.J.H. has no competing interest.

T.G. has no competing interest.

D.A. has no competing interest.

C.G. has no competing interest.

Y.B.‐S. has no competing interest.

H.Z. has served at scientific advisory boards for AbbVie, Alector, Eisai, Denali, Roche Diagnostics, Wave, Samumed, Siemens Healthineers, Pinteon Therapeutics, Nervgen, AZTherapies, and CogRx; has given lectures in symposia sponsored by Cellectricon, Fujirebio, Alzecure, and Biogen; and is a cofounder of Brain Biomarker Solutions in Gothenburg AB (BBS), which is a part of the GU Ventures Incubator Program (outside the submitted work).

D.G.G. has received honoraria from Bial Pharma, GE Healthcare, and Vectura plc and consultancy fees from the Glasgow Memory Clinic.

H.R.M. is employed by UCL. In the past 24 months he reports paid consultancy from Biogen, UCB, AbbVie, Denali, Biohaven, and Lundbeck; lecture fees/honoraria from Biogen, UCB, C4X Discovery, GE‐Healthcare, Wellcome Trust, and Movement Disorders Society; and research grants from ASAP, Parkinson's UK, Cure Parkinson's Trust, PSP Association, CBD Solutions, Drake Foundation, and Medical Research Council. Dr. Morris is a co‐applicant on a patent application related to C9ORF72—Method for diagnosing a neurodegenerative disease (PCT/GB2012/052140).

T.F. has received grants from the National Institute of Health Research, The Michael J. Fox Foundation, John Black Charitable Foundation, Cure Parkinson's Trust, Innovate UK, Van Andel Research Institute, and Defeat MSA. He has served on advisory boards for Voyager Therapeutics, Handl Therapeutics, Living Cell Technologies, Bial, and Profile Pharma. He has received honoraria for talks sponsored by Bial, Profile Pharma, and Boston Scientific.

## Author Roles

1. Research project: A. Conception, B. Organization, C. Execution;
2. Manuscript preparation: A. Writing of the first draft, B. Review and critique.

N.V.: 1A, 1B, 1C, 2A, 2B

M.L.: 1C, 2B

R.R.: 1C, 2B

A.J.H.: 1C, 2B

T.G.: 1C, 2B

D.A.: 1C, 2B

C.G.: 1C, 2B

Y.B.‐S.: 1C, 2B

H.Z.: 1C, 2B

D.G.G.: 1A, 1B, 1C, 2B

H.R.M.: 1A, 1B, 1C, 2B

T.F.: 1A, 1B, 1C, 2B.

## Data Availability

The data that support the findings of this study are available on request from the corresponding author. The data are not publicly available due to privacy or ethical restrictions.
